# KIF18B promotes tumor progression in osteosarcoma by activating β-catenin

**DOI:** 10.20892/j.issn.2095-3941.2019.0452

**Published:** 2020-05-15

**Authors:** Tian Gao, Ling Yu, Zhiwei Fang, Jiayong Liu, Chujie Bai, Shu Li, Ruifeng Xue, Lu Zhang, Zhichao Tan, Zhengfu Fan

**Affiliations:** ^1^Key Laboratory of Carcinogenesis and Translational Research (Ministry of Education/Beijing), Department of Bone and Soft Tissue Tumor, Peking University Cancer Hospital & Institute, Beijing 100142, China; ^2^Department of Orthopedics, Renmin Hospital of Wuhan University, Wuhan 430060, China

**Keywords:** β-catenin, APC, ATF2, KIF18B, osteosarcoma

## Abstract

**Objective:** Osteosarcoma is a common primary highly malignant bone tumor. Kinesin family member 18B (*KIF18B*) has been identified as a potential oncogene involved in the development and metastasis of several cancer types. While KIF18B overexpression in osteosarcoma tissue is clearly detected, its specific function in the disease process remains to be established.

**Methods:** *KIF18B* expression was assessed in osteosarcoma tissues and cells. We additionally evaluated the effects of KIF18B on proliferation, migration, and invasion of osteosarcoma cells, both *in vitro* and *in vivo*.

**Results:** Our results showed overexpression of *KIF18B* in osteosarcoma tissues and cells. Knockdown of *KIF18B* induced G1/S phase arrest and significantly inhibited proliferation, migration, and invasion of osteosarcoma cells, both *in vitro* and *in vivo*. *KIF18B* regulated β-catenin expression at the transcriptional level by controlling nuclear aggregation of ATF2 and at the post-transcriptional level by interacting with the adenomatous polyposis coli (*APC*) tumor suppressor gene in osteosarcoma cells.

**Conclusions:** KIF18B plays a carcinogenic role in osteosarcoma by regulating expression of β-catenin transcriptionally *via* decreasing nuclear aggregation of ATF2 or post-transcriptionally through interactions with APC. Our collective findings support the potential utility of KIF18B as a novel prognostic biomarker for osteosarcoma.

## Introduction

Osteosarcoma, a malignancy derived from mesenchymal tissue, is the second most prevalent type of primary bone tumor^1^. The majority of osteosarcomas are highly malignant and most patients clinically diagnosed with osteosarcoma have metastatic disease at initial presentation^2^. Owing to the growth and invasiveness of osteosarcoma, a periosteal reaction occurs in a radial manner. Osteosarcoma is particularly prone to early metastasis to the lung, which accounts for low annual survival rates of 5%–20% post-amputation^3^, highlighting the urgent requirement for novel molecular biomarkers to facilitate earlier diagnosis, accurately predict clinical outcomes, and promote effective treatments.

The classical Wnt/β-catenin signaling pathway is involved in the regulation of multiple biological processes, including embryogenesis and morphogenesis, tissue stability, energy metabolism balance, and stem cell maintenance. Dysregulation of this pathway is often closely associated with major human diseases. β-Catenin protein is highly expressed in almost all tumorigenesis processes, resulting in abnormal activation of Wnt/β-catenin signaling and consequent promotion of cell colonization and migration, effects on the normal cell cycle, and induction of tumorigenesis and deterioration^4–7^. Therefore, therapies targeting Wnt/β-catenin signaling present a promising anti-cancer strategy.

KIF18B is a member of the kinesin superfamily that is closely associated with the pairing and separation of chromosomes in mitosis and controls microtubule length, centering the mitotic spindle at metaphase^8–10^. The kinesin superfamily comprises a conserved class of microtubule-dependent molecular motor proteins with adenosine triphosphatase activity^11,12^. According to the standard nomenclature for kinesin, 45 KIFs have been identified and classified into 14 families (kinesin-1 to kinesin-14)^11,13^. These families are subdivided into N-kinesin, M-kinesin, and C-kinesin, based on location of their kinematic domains at the amino, middle, and carboxy terminus, respectively^14^. During eukaryotic mitosis, KIFs are involved in spindle formation, chromosome recombination and alignment, and cytokinesis^15^. Abnormal KIF expression and function are implicated in the development of multiple human cancer types. Higher recovery of KIF function may therefore be beneficial in molecular targeted therapies for human cancers^16^. Assessment of KIF expression patterns may provide clues for biomarkers suitable for early detection and prognosis of human cancers. For instance, *KIF5B* mRNA is upregulated in several cancer tissues, including bladder, stomach, skin, and breast^17–20^. Inactivation of the KAP3 subunit of the KIF3 complex in neural precursor cells promotes embryonic brain tumors^21^. KIF14, a microtubule motor, is overexpressed in breast and lung cancers and retinoblastoma^22,23^. KIF18A is highly expressed in most cancer cells but not paracarcinoma tissue, except testes and lungs, as reported by Shichijo et al*.*^24^ Bioinformatic analyses suggest that overexpression of KIF18B is associated with poor prognosis in hepatic carcinoma^25^. Earlier, Wu and co-workers^26^ characterized *KIF18B* as a novel oncogene that promotes tumorigenicity of cervical cancer cells through activating the Wnt/β-catenin signaling pathway. Experiments by our group showed KIF18B overexpression in osteosarcoma tissue, but its specific function in the disease process is currently unclear. Data from the present study suggest that *KIF18B* is a potential oncogene that promotes osteosarcoma cell proliferation and migration, both *in vitro* and *in vivo*. Furthermore, KIF18B exerts its effects through regulation of β-catenin, a key protein in the Wnt/β-catenin pathway, at both transcriptional and post-transcriptional levels.

## Materials and methods

### Cell culture

Human osteoblast cells (hFOB1.19) and three sarcoma cell lines (HOS, U2OS, and Saos-2) were obtained from American Type Culture Collection (ATCC, Manassas, VA, USA). All cell lines were cultured in DMEM supplemented with 10% fetal bovine serum (ProSpec-Tany TechnoGene, Ltd., Ness-Ziona, Israel) and 100 mg/mL penicillin/streptomycin (ProSpec-Tany TechnoGene, Ltd.). Cultures were additionally supplemented with 200 mM/L glutamine and 2 mg/mL sodium bicarbonate for hFOB1.19 cells, 1.0 g/L glucose and 1 mM sodium pyruvate for HOS and U2OS cells, and 2 mM L-glutamine for Saos-2 cells. Cells were grown in a humidified 5% CO_2_ atmosphere at 37 °C.

### Cell transfection

Human *KIF18B*-targeting small hairpin RNA (shRNA1) sequences were as follows: 5′-GCUCAUCAACGUCCUCAAUTT-3′ (sense) and 5′-AUUGAGGACGUUGAUGAGCTT-3′ (antisense)^26^. Human *KIF18B*-targeting small hairpin RNA (shRNA2) was purchased from Sigma (St. Louis, MO, USA) (TRCN0000245670). Recombinant *KIF18B* or negative control (shRNA-KIF18B or shRNA-NC) lentiviral particles were generated for transfection of cells. *ATF2* siRNA (106604), *CUX1* siRNA (110461), *TCF3* siRNA (114338), *RUNX1* siRNA (106565), *GATA1* siRNA (157704),* APC* siRNA (122389) and control siRNA (131905) were purchased from Invitrogen (Carlsbad, CA, USA). Plasmids expressing full-length human KIF18B and β-catenin protein were purchased from Genechem (Shanghai, China). Empty plasmid was used as the negative control. *KIF18B* and *CTNNB1* cDNA were cloned into pcDNA3.1 (GENEray Biotechnology, Shanghai, China) for constructing overexpression plasmids, with empty vector used as a negative control. Transfection was performed using Oligofectamine (Invitrogen Life Technologies, Carlsbad, CA, USA), in keeping with the manufacturer’s instructions. Transfection efficiency was detected *via* quantitative real-time reverse transcription-polymerase chain reaction (qRT-PCR).

### RNA extraction and qRT-PCR

Osteoblasts hFOB1.19 and osteosarcoma cells were harvested and collected, and total RNA extracted with TRIzol reagent (Thermo Fisher Scientific Inc., Waltham, MA, USA). The quality and quantity of RNA were assessed using a NanoDrop 2000 spectrophotometer (Thermo Fisher Scientific Inc.). For qRT-PCR, a reverse transcription kit (Cat: RR036A; Takara, Japan) was employed. The reaction was performed with 1,000 ng total RNA and SYBR Select Master Mix (Cat: 4472908; Applied Biosystems, Foster, CA, USA) in a final volume of 20 μL. The primers used for *KIF18B*, *CTNNB*, *WNT5A*, *MYC*, *CCND1*, *ATF2*, *CUX1*, *TCF3*, *RUNX1*, *GATA1* and β-*actin* genes are listed in **Supplementary Table S1**. The QuantStudio™ 6 Flex Real-Time PCR System was employed for collection of qRT-PCR data. The qRT-PCR reaction included an initial denaturation step at 95 °C for 10 min, followed by 40 cycles at 92 °C for 15 s, and 60 °C for 1 min. Experiments were performed in triplicate and relative expression calculated and normalized to β-actin using the 2^−ΔΔCt^ method.

### Western blot

Cells were harvested and processed in lysis buffer (Tris-HCl, sodium dodecyl sulfate (SDS), β-mercaptoethanol, and glycerol) on ice. A BCA Kit (KeyGEN, Pierce, USA) was utilized for protein quantitation. Equal amounts of protein were separated *via* SDS-polyacrylamide gel electrophoresis and transferred to polyvinylidene fluoride (PVDF) membranes. Next, membranes were blocked in 5% skimmed milk powder in Tris-buffered saline/Tween 20 (TBS-T) for 1 h and incubated overnight at 4 °C with primary antibodies against KIF18B (ab168812, 1:2,000), β-catenin (ab227499, 1:1,000), Wnt5a (ab174963, 1:500), Myc (ab32072, 1:1,000), cyclin D1 (ab16663, 1:200), ATF2 (ab47476, 1:1,000), DDK (ab1162, 1:5,000), β-tubulin (ab210797, 1:1,000), Lamin B1 (ab133741, 1:2,000), β-actin (ab179467, 1:5,000), APC (ab15270, 1:5,000) or GAPDH (ab181602, 1:10,000). After washing with TBS-T, membranes were incubated with goat anti-rabbit secondary antibody (ab7090, 1:1 00) at room temperature for 2 h. The primary and secondary antibodies were purchased from Abcam (Cambridge, MA, USA). Blots were visualized *via* enhanced chemiluminescence detection (Thermo Fisher Scientific Inc., Waltham, MA, USA). All experiments were independently repeated at least three times.

### Colony forming assay

Cell proliferation was examined using Cell Counting Kit-8 (CCK-8) (KeyGEN). Transfected cells and controls were seeded at densities of 5,000 and 10,000 cells per well, and absorbance measured at 450 nm using an ELx-800 universal microplate reader (BioTek, CA, USA). Cells were grown for one week, with changing of medium every second day. Cells were subsequently fixed with 4% paraformaldehyde and stained with 0.1% crystal violet for analysis. The visible colonies were counted. Each experiment was performed in triplicate.

### Cell migration and invasion assays

Cell migration and invasion assays were performed in 6.5 mm Transwells (CAS No. 3422, Life Sciences, PO, USA). Cells (5 × 10^4^) in 100 μL serum-free medium were added to the upper chamber and the lower chamber filled with complete medium containing 10% serum. After 48 h of incubation at 37 °C and 5% CO_2_, non-migrating cells present on the upper surface were removed. Membranes were fixed in 4% paraformaldehyde and stained with 0.1% crystal violet. The number of migrating cells from four random fields was evaluated under a microscope. Each sample was assayed in triplicate. A similar system with Matrigel-coated membranes was employed for assessing invasion. In this case, Transwells were first coated with Matrigel (BD Biosciences NY, USA) and cells were allowed to invade for 72 h.

### Flow cytometry analysis

Flow cytometry was applied to determine cell cycle distribution and apoptosis. For the former analysis, cells were harvested and fixed with 70% ethanol at 20 °C for 24 h, followed by the addition of propidium iodide solution into cell suspensions at a final concentration of 100 μg/mL. After incubation for 10 min in the dark, cells were washed with PBS containing 0.5% BSA. Samples were analyzed using a FACSCalibur flow cytometer (Beckman, CA, USA). The percentages of cells in G0–G1, S, and G2–M phases were compared. Cells were washed and resuspended in PBS buffer, and the Annexin V-FITC cell apoptosis detection kit used for analysis of apoptosis according to the manufacturer’s instructions (BD Biosciences). Following incubation at room temperature in the dark for 20 min, cells were immediately analyzed *via* flow cytometry using FACScan. All samples were examined in triplicate.

### Plasmid transfection and luciferase reporter assays

The day prior to transfection, U2OS cells were suspended in fresh medium and plated in 96-well plates at a density of 2 × 10^4^ cells/well. Upon reaching 70% confluency, cells were transfected with 50 ng pGL3-Basic-CTNNB1 promoter and 0.5 ng pRL-TK (internal control), along with 50 ng pGL3-Basic-CTNNB1 (mutant) promoter using the Lipofectamine™ 2000 Transfection Reagent (Invitrogen Life Technologies), in keeping with the manufacturer’s instructions. The total amount of plasmid DNA was maintained as 200 ng in each well. Cells were harvested and lysed at 24 h post-transfection. Firefly and renilla luciferase activities were determined with the Dual-Luciferase Reporter Assay System in a GloMax96 luminescence reader (Promega, Madison, WI, USA) according to the manufacturer’s protocol. Relative luciferase activity was expressed as the ratio of firefly luciferase to renilla luciferase activity in each sample. All values were obtained from at least three independent repeat transfections, with six wells for each transfection mixture/sample.

### Co-immunoprecipitation assay

Cells were harvested with 1% NP40 lysis buffer containing protease and phosphatase inhibitors [150 mM NaCl, 50 mM Tris-HCl (pH7.4) and 1% Nonidet P-40]. The supernatant was removed *via* centrifugation at 1,000 × *g* for 1 min. ATF2, DDK, APC or β-catenin antibodies bound to protein A/G agarose using the Protein G immunoprecipitation kit (Sigma-Aldrich, cat. 11719386001). The agarose slurry (20 μL) was washed twice with 200 μL PBS buffer solution and incubated with 100 μL antibody (10 μL antibody + 85 μL H_2_O + 5 μL 20 × PBS) in PBS for 30 min at 25 °C on a mixer. In parallel, 100 μL rabbit serum with the same IgG concentration or anti-rabbit IgG peroxidase secondary antibody was prepared as a negative control. Agar glycoprotein and 10 μL antibody were incubated in a mixer for 1 h at 25 °C, followed by overnight incubation with 600 μL pre-clarified lysates. Immunoprecipitation products were washed five times with washing buffer and eluted with 2× Laemmli buffer at 100 °C for 10 min. Three independent experiments were conducted.

### Chromatin immunoprecipitation

Cells (1 × 10^7^) grown in 150 mm dishes were fixed with 1/10 volume of cross-linking solution [11% formaldehyde/0.1 mol/L NaCl/1 mmol/L Na-EDTA/0.5 mmol/L Na-EGTA/50 mmol/L HEPES (pH 8.0)] for 10 min at 37 °C. Chromatin immunoprecipitation (ChIP) analysis was performed as reported previously^27^. qPCR primers for the CTNNB1 promoter regions are listed in **Supplementary Table S1**.

### *In vivo* experiments

Ten nude mice (aged 4–6 weeks, IACUC No. 2019YZJ78) purchased from the Baylor College of Medicine Institutional Animal Care and Use Committee were subcutaneously injected in the axilla region with exponentially growing U2OS cells with fluorescent tag (1.0 × 10^6^) transfected with shKIF18B or shCtrl. Tumor volumes were measured every 7 days using calipers. Approximately six weeks later, mice were anesthetized with 1% sodium pentobarbital and intraperitoneally injected with luciferin (10 μL/g). Tumor volumes were measured and tumors collected for immunohistochemical (IHC) examination.

### Immunohistochemistry

Paraffin sections (7 μm) were incubated in methanol/H_2_O_2_ (30 min) to inhibit endogenous peroxidase activity, washed with PBS for 5 min, and blocked with normal rabbit serum for 20 min at room temperature (RT). Incubation with anti-PTEN (ab170941, Abcam, 1:100), anti-β-catenin (ab6302, Abcam, 1:2,000), anti-Ki67 (ab16667, Abcam, 1:1,000) and anti-KIF18B (ab121798, Abcam, 1:200) was performed overnight at 4 °C. Sections were subsequently incubated with the biotinylated secondary antibody (1 h, RT) and avidin-conjugated peroxidase (45 min, RT). Between each step, sections were washed three times with PBS. The peroxidase staining reaction was conducted with diaminobenzidine (1 mg/mL) and H_2_O_2_ for 5 min and terminated by immersion in tap water for 10 min. Sections were counterstained with hematoxylin for 1 min. In control groups, the primary antibody was replaced with pre-immune mouse serum. Both positive and negative controls were included for all assays.

### Statistical analysis

All statistical analyses were conducted using the SPSS 18.0 statistical software package (SPSS Inc., USA). Values are expressed as means ± standard deviation. The Mann-Whitney U test was applied for comparison of two groups of independent data. The Kolmogorov-Smirnov test was used for statistical analysis of the normality of parameter distribution. *P* values < 0.05 were considered statistically significant.

## Results

### KIF18B is overexpressed in osteosarcoma tissues and cells

**Figure 1:**
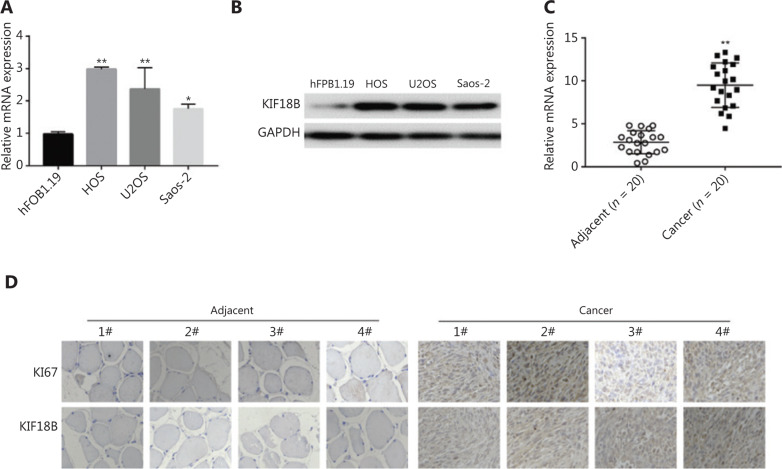
*KIF18B* is overexpressed in osteosarcoma tissues and cells. (A) mRNA and (B) protein expression of *KIF18B* was higher in the three osteosarcoma cell lines than in hFOB1.19. The Mann-Whitney U test was used for statistical analysis. *KIF18B* mRNA expression in the osteosarcoma cell lines (HOS, U2OS, and Saos-2) was significantly higher than that in hFOB1.19 immortalized human osteoblasts, as determined *via* RT-qPCR using GAPDH as a reference gene (*n* = 3). Highest expression of KIF18B was detected in HOS cells (three times that in hFOB1.19). KIF18B protein expression in HOS, U2OS, and Saos-2 cell lines was significantly higher than that in hFOB1.19, consistent with mRNA results. (C) *KIF18B* is overexpressed in osteosarcoma tissues of patients. Higher *KIF18B* expression was detected in tumor tissues of 75% (15/20) patients. The Mann-Whitney U test was used for statistical analysis. (D) Immunohistochemical staining for KIF18B in cancer and adjacent tissues from patients with osteosarcoma.

Differential mRNA and protein expression of *KIF18B* in immortalized human osteoblasts, hFOB1.19, and three sarcoma cell lines, HOS, U2OS, and Saos-2, was detected with qRT-PCR and Western blot, respectively. *KIF18B* mRNA expression was 2–3 times higher in osteosarcoma cells than hFOB1.19 cells (**Figure 1A**). Consistently, KIF18B protein was overexpressed in all three osteosarcoma cell lines, in particular, HOS and U2OS (**Figure 1B**). Further qRT-PCR analysis of *KIF18B* in 20 paired osteosarcoma tissue samples (tumor and adjacent normal tissues) revealed overexpression in malignant tissue in 75% (15/20) cases (*P* < 0.001; **Figure 1C**). The results of immunohistochemical staining for KIF18B and Ki67 of cancer tissue and adjacent tissue of osteosarcoma patients were analyzed, reveled higher expression of Ki67 and KIF18B in tissue of osteosarcoma compared to that of adjacent tissue (**Figure 1D**).

### Knockdown of KIF18B affects proliferation, migration, and invasion of osteosarcoma cells

**Figure 2:**
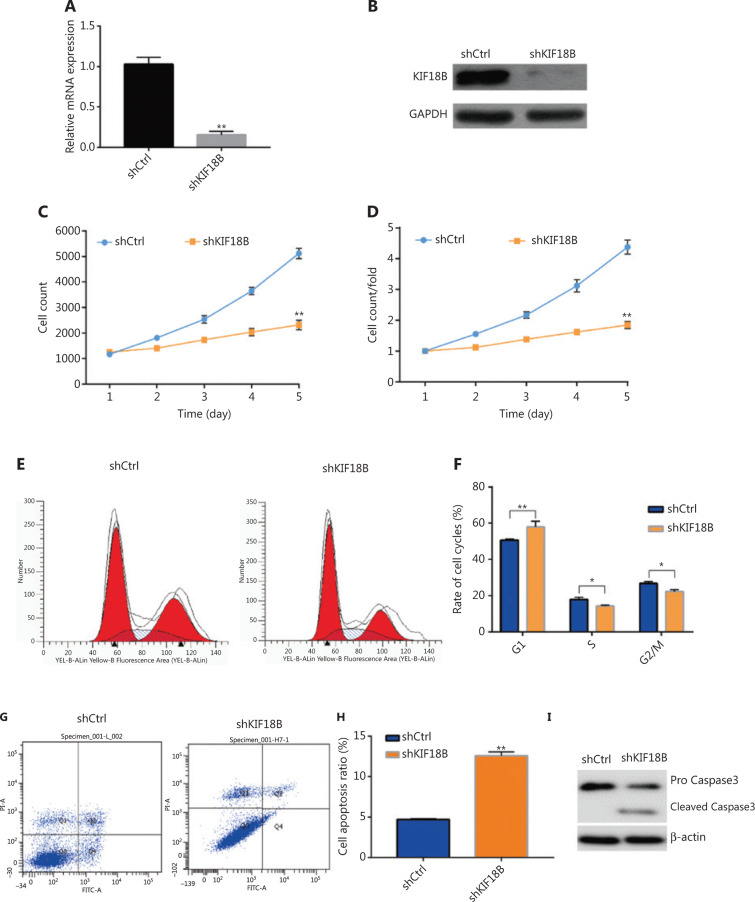
Knockdown of *KIF18B* affects proliferation of osteosarcoma cells. (A) mRNA and (B) protein expression of *KIF18B* was downregulated in the shKIF18B-transfected cell line. The *KIF18B* mRNA level in the shKIF18B group was 18% that in the shCtrl group, as determined using qPCR. The protein level was < 10% that in the shCtrl group, as determined using Western blot. (C) Cell count curves. (D) Cell count fold changes between shCtrl and shKIF18B transfection groups. (E) Distribution of cell cycle phases for shCtrl and shKIF18B cells, determined *via* flow cytometry. (F) Proportion of each cell cycle phase in shCtrl and shKIF18B cell groups. Compared to shCtrl cells, KIF18B knockdown induced a significant increase in cells in the G1 phase and decrease in cells in S and G2/M phases. (G) *KIF18B* knockdown enhanced apoptosis in U2OS cells. (H) Proportion of apoptotic cells in shCtrl and shKIF18B lines. The apoptosis rate was 12.5% in shKIF18 cells and 4.7% in shCtrl cells. (I) Western blot analysis of activation of Caspase 3. Pro Caspase3 was expressed in both shCtrl and shKIF18B cell lines and cleaved Caspase 3 detected in the shKIF18B cell line. The Kolmogorov-Smirnov test was used for statistical analysis of data from C and D and Mann-Whitney U test for data from A, F and H.

Two shRNA lentiviral vectors specifically targeting *KIF18B* were constructed for infection of U2OS and HOS cells after viral packaging. The *KIF18B* mRNA level in the shKIF18B (shKIF18B#1, denoted shKIF18B) group was 18% that in the U2OS shCtrl group (**Figure 2A**), and the protein level was < 10% that in the shCtrl group (**Figure 2B**), according to qRT-PCR and Western blot analyses, respectively. The *KIF18B* mRNA level in the shKIF18B (shKIF18B#2) group was 21% relative to the U2OS shCtrl group (**Supplementary Figure S2A**) and the protein level was < 13% that in the shCtrl group (**Supplementary Figure S2B**). Consistent results were obtained with the HOS cell line. *KIF18B* mRNA levels in the shKIF18B#3 and shKIF18B#4 groups were 26% and 27% (**Supplementary Figure S2A**) and the corresponding protein levels were < 15% and 17% relative in the HOS shCtrl group (**Supplementary Figure S2B**), respectively.

With prolonged culture, the differences between the number of cells in the shKIF18B and control groups gradually became clear. By day 5, the shKIF18B cell number was only a third of the control group (**Figure 2C and 2D**). To gain further insights into the effects of KIF18B suppression in U2SO cells, cell cycle distribution was analyzed. *KIF18B* knockdown led to alterations in the periodic distribution of U2OS cells. Compared to shCtrl cells, cells depleted of *KIF18B* were significantly increased in the G1 phase and decreased in the S and G2/M phases (**Figure 2E and 2F**), indicating that downregulation of KIF18B induces G1/S phase arrest in U2OS cells. Furthermore, KIF18B knockdown promoted apoptosis of U2OS cells (**Figure 2G and 2H**).

**Figure 3:**
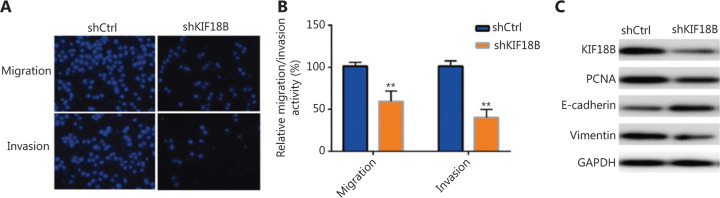
Knockdown of *KIF18B* affects migration and invasion of osteosarcoma cells. (A) Transwell assays of shCtrl and shKIF18B cell lines. Knockdown of KIF18B impaired cell migration and invasion ability. (B) Relative migration and invasion activities of shCtrl and shKIF18B cell lines. The Mann-Whitney U test was used for statistical analysis. Migration and invasion abilities of shKIF18B cells were 41.5% and 60.2% relative to shCtrl cells, respectively. (C) Expression analysis of proteins associated with the EMT. Compared with shCtrl cells, shKIF18B cells contained higher levels of E-cadherin and lower levels of vimentin and PCNA.

Transwell assays were additionally performed to investigate the effects of KIF18B on U2OS cell migration and invasion. As shown in **Figure 3A**, migration and invasion of cell groups transfected with shCtrl and shKIF18B from the upper to the lower chamber were distinct. Penetration of shKIF18B-transfected cells through the Matrigel to the lower surface of the filter was inhibited (**Figure 3B**), suggesting that knockdown of KIF18B attenuates the U2OS migration and invasion. Further examination of EMT-associated proteins revealed that compared with shCtrl cells, the shKIF18B cells contained lower E-cadherin and higher vimentin levels, supporting a positive correlation between expression of KIF18B and EMT (**Figure 3C**). Similar results were obtained with the HOS cell line (**Supplementary Figure S2C**).

### Knockdown of KIF18B suppresses migration and invasion of osteosarcoma cells through effects on β-catenin expression

**Figure 4:**
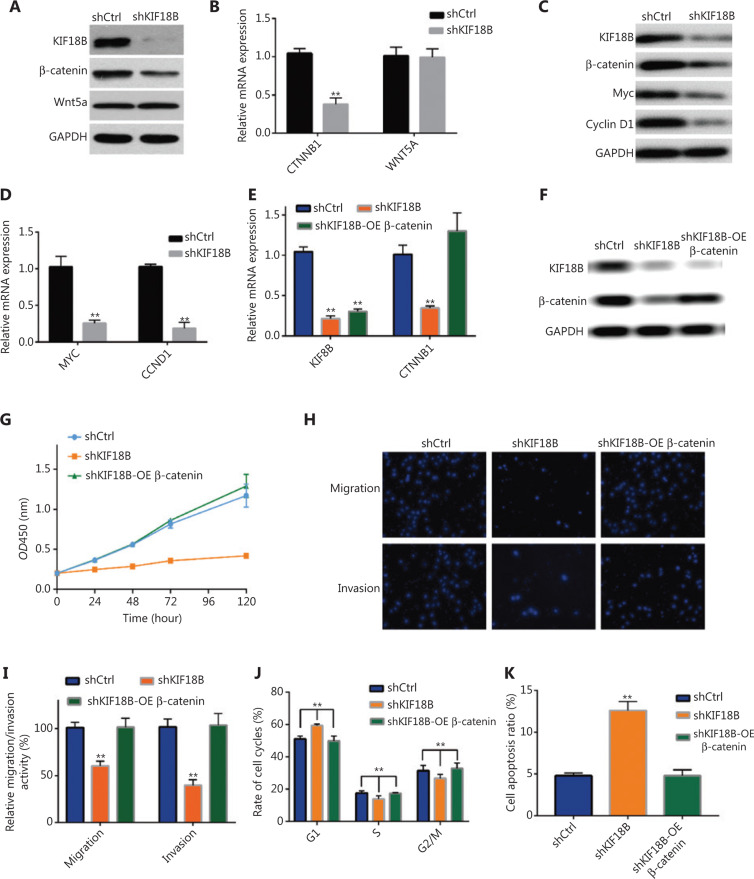
Knockdown of *KIF18B* suppresses migration and invasion of osteosarcoma cells through effects on β-catenin expression. Protein and mRNA expression were analyzed *via* qPCR and Western blot, respectively. (A) Protein and (B) mRNA expression of β-catenin and Wnt5a in shCtrl and shKIF18B cell lines. *KIF18B* knockdown resulted in lower mRNA and protein levels of CTNNB1 than shCtrl treatment whereas expression of WNT5A was not affected. (C) Protein and (D) mRNA expression of Myc, and cyclin D, genes downstream of *CTNNB1*, in shCtrl and shKIF18B cell lines. Protein levels of CTNNB1 (β-catenin), c-MYC, and cyclin D1 were significantly downregulated and the mRNA levels of c-MYC and cyclin D1 decreased to 25.1% and 17.9% in shKIF18B cell lines, respectively. (E) Protein and (F) mRNA expression of KIF18B and β-catenin in shCtrl, shKIF18B, and shKIF18B-OE β-catenin cell lines. Expression of KIF18B in shKIF18B and shKIF18B-OE β-catenin cells was significantly downregulated. Expression of CTNNB was recovered in shKIF18B-OE β-catenin cells, but remained very low in shKIF18B cells. (G) CCK-8 analysis of shCtrl, shKIF18B, and shKIF18B-OE β-catenin cell lines. shKIF18B-OE β-catenin cells showed recovery of proliferative activity to control levels. (H) Transwell assays of shCtrl, shKIF18B, and shKIF18B-OE β-catenin cell lines. (I) Relative migration and invasion activities of shCtrl, shKIF18B, and shKIF18B-OE β-catenin cell lines. Migration and invasion activities of shKIF18B-OE β-catenin cells were recovered to control levels. (J) Proportion of shCtrl, shKIF18B, and shKIF18B-OE β-catenin cells in each cell cycle phase. The proportion of cells in each cell cycle phase in shKIF18B-OE β-catenin cells was similar to that of the control group. (K) Apoptotic cell ratios in shCtrl, shKIF18B, and shKIF18B-OE β-catenin lines. The apoptosis ratio in shKIF18B-OE β-catenin cells was similar to that of the control group. The Mann-Whitney U test was used for statistical analysis in B, E, and I–K and Kolmogorov-Smirnov test applied for G.

To establish the specific role of KIF18B in progression of osteosarcoma, differentially expressed genes between shCtrl and shKIF18B cells were detected *via* RNA-seq. Among the top 15 genes downregulated in the two cell lines (**Supplementary Table S3**), we identified *CTNNB1* and *WNT5A*, two key genes participating in the Wnt classical and non-canonical pathways, respectively. Accordingly, we speculated that KIF18B may affect the tumorigenicity of osteosarcoma cells by influencing the Wnt signaling pathway. To examine this hypothesis, expression of *CTNNB1* and *WNT5A* in shKIF18B cells was examined *via* qRT-PCR and Western blot. In agreement with results of enrichment analyses, *KIF18B* knockdown resulted in lower mRNA (**Figure 4A**) and protein (**Figure 4B**) levels of *CTNNB1* than shCtrl treatment whereas expression of *WNT5A* was not affected. Examination of c-MYC and cyclin D1 genes downstream of CTNNB1 (β-catenin) additionally revealed significant downregulation of both mRNA (**Figure 4C**) and protein (**Figure 4D**) levels. Notably, CTNNB1 overexpression in shKIF18B cell lines (**Figure 4E, 4F**) resulted in recovery of proliferation (**Figure 4G**), migration, invasion, proportion of cells in each cell cycle phase, and proportion of apoptotic cells to control levels (**Figure 4H–4K**). These results clearly suggest that the function of KIF18B in osteosarcoma cells is closely associated with activation of β-catenin, a key member of the Wnt/β-catenin pathway.

### KIF18B regulates β-catenin at the transcriptional level

**Figure 5:**
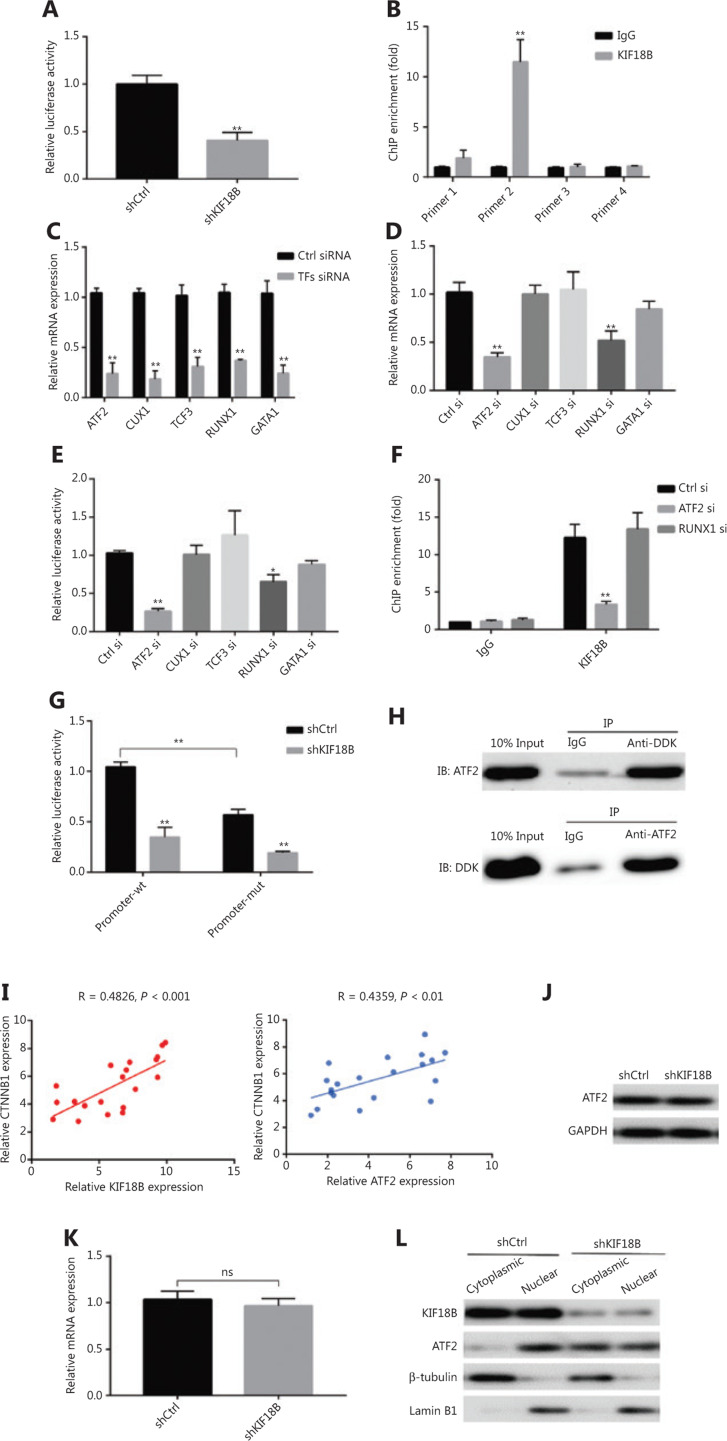
KIF18B regulates β-catenin at the transcriptional level through ATF2. (A) Relative luciferase activity was determined in U2OS cells co-transfected with *CTNNB1* promoter-luciferase reporter and shKIF18B or a non-targeting control vector (shCtrl). Upon *KIF18B* knockdown, luciferase activity was significantly decreased. (B) ChIP-qPCR enrichment for quantification of binding to the *KIF18B* locus. The *CTNNB1* promoter was subdivided evenly into four sections. The second section exhibited binding to the promoter region of β-catenin. (C) qPCR analysis of mRNA expression of TFs that bind the promoter region of β-catenin. (D) mRNA expression of *CTNNB1* in five TF siRNA cell lines. Downregulation of ATF2 and RUNX1 affected *CTNNB1* mRNA expression*.* (E) Relative luciferase activity was determined in five TF siRNA cells co-transfected with CTNNB1 promoter-luciferase reporter and shKIF18B or a non-targeting control vector (shCtrl). The luciferase activities of the promoter-reporter vector in the ATF2 siRNA groups were downregulated to different degrees. (F) ChIP-qPCR enrichment to quantify binding to the *KIF18B* locus in Ctrl siRNA, ATF2 siRNA, and RUNX1 siRNA cells. KIF18B binding to the promoter region was significantly downregulated only when ATF2 expression was decreased. (G) Determination of relative luciferase activity in U2OS cells co-transfected with *CTNNB1* promoter-wt and *CTNNB1* promoter-mut-luciferase reporter and shKIF18B or a non-targeting control vector (shCtrl). Upon deletion of the potential ATF2 binding site in the promoter region of β-catenin, KIF18B significantly suppressed transcriptional activation of luciferase. (H) Interactions between KIF18B and ATF2 in U2OS cells overexpressing *KIF18B* with a DDK tag, detected *via* co-immunoprecipitation. KIF18B interacted with ATF2. (I) qPCR analysis of co-expression of β-catenin and KIF18B or ATF2. A positive relationship between co-expression of β-catenin, KIF18B and ATF2 was evident. (J) Protein and (K) mRNA expression of *ATF2* in shCtrl and shKIF18B cell lines. Knockdown of KIF18B did not affect mRNA and protein expression levels. (L) Protein expression of KIF18B and ATF2 in cytoplasmic and nuclear fractions of shCtrl and shKIF18B cell lines. Nuclear distribution of ATF2 was significantly decreased in shKIF18B cell lines. The Mann-Whitney U test was used for statistical analysis in (A–H, K).

Studies to date have shown β-catenin regulation at the protein rather than transcript level. However, knockdown and overexpression of *KIF18B* additionally affected the relative β-catenin mRNA content in the current study. The promoter region of β-catenin was amplified using PCR and used to construct a luciferase reporter vector. Upon *KIF18B* knockdown, luciferase activity was significantly decreased, suggesting that KIF18B acts on the promoter region and positively regulates transcription of β-catenin (**Figure 5A**). Data from ChIP experiments further validated binding of KIF18B to β-catenin promoter (**Figure 5B**). No previous studies have described a DNA binding domain although an ATP-binding domain of KIF18B has been identified, highlighting the possibility that KIF18B interacts indirectly with the promoter of β-catenin. Next, we used the online tools PROMO (http://alggen.lsi.upc.es/cgibin/promo_v3/promo/promoinit.cgi?dirDB=TF_8.3) and TRANSFAC (http://gene-regulation.com/pub/databases.html) to predict transcription factors that potentially bind the β-catenin promoter. ATF-2 (ATF2), CUTL1 (CUX1), E47 (TCF3), Evi-1 (RUNX1), and GATA-1 (GATA1) were the top ranked hits. Upon utilization of siRNAs to suppress the levels of these transcription factors, mRNA expression of β-catenin and luciferase activity of the promoter-reporter vector in the ATF2 and RUNX1 siRNA groups showed different degrees of downregulation (**Figure 5C–5E**). ChIP experiments confirmed that KIF18B binding at the promoter region was significantly decreased, along with a specific decrease in ATF2 expression (**Figure 5F**). After deletion of the potential binding site of ATF2 at the promoter region of β-catenin in the luciferase reporter vector (**Supplementary Figure S2**), KIF18B induced significant downregulation of the transcriptional activation of luciferase (**Figure 5G**). Our results suggest that KIF18B interacts with the promoter region of β-catenin *via* ATF2. In osteosarcoma specimens, qPCR analysis showed positive co-expression of β-catenin with KIF18B and ATF2 relative to that in adjacent tissues (**Figure 5I**). Immunoprecipitation assays (Co-IP) performed on U2OS cells overexpressing *KIF18B* with a DDK tag revealed interactions between KIF18B and ATF2 (**Figure 5H**). Knockdown of *KIF18B* did not affect the mRNA and protein expression levels of *ATF2* (**Figure 5J and 5K**) but altered nuclear distribution and decreased nuclear aggregation of ATF2 to a significant extent (**Figure 5L**). Kinesin is based on cytoskeleton components, such as microtubules, and transports different molecular cargo proteins to designated intracellular locations. As a member of the kinesin family, KIF18B may be contribute to regulation of β-catenin transcription by transporting ATF2 into the nucleus.

### KIF18B regulates β-catenin at the post-transcriptional level

**Figure 6:**
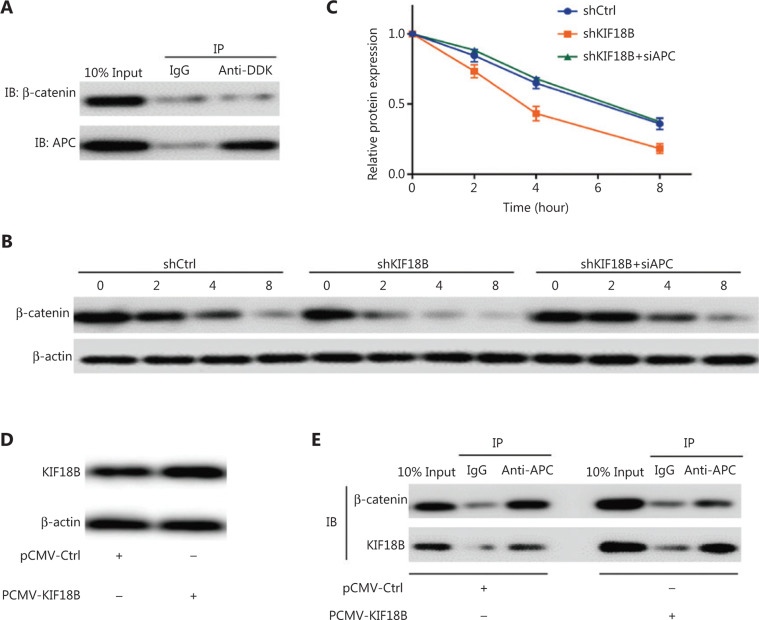
KIF18B regulates β-catenin at the post-transcriptional level through interactions with APC. (A) Interactions between KIF18B and ATF2 in U2OS cells overexpressing *KIF18B* with a DDK tag detected *via* co-immunoprecipitation. KIF18B did not interact with β-catenin. (B, C) Protein expression of β-catenin in shCtrl and shKIF18B cells under cycloheximide treatment. Protein stability of β-catenin in the shKIF18B group was significantly lower than that in the shCtrl group whereas in the shKIF18B group with APC downregulation, β-catenin stability was recovered to a similar level as that in the shCtrl group. The Kolmogorov-Smirnov test was used for statistical analysis in (C). (D) Overexpression of KIF18B in cell lines infected with pCMV-KIF18B. (E) Interactions between KIF18B or β-catenin and APC in U2OS cells overexpressing KIF18B or Ctrl, detected *via* co-immunoprecipitation. Overexpression of KIF18B significantly blocked interactions of APC with β-catenin.

Co-IP experiments revealed no interactions between KIF18B and β-catenin directly (**Figure 6A**). Protein stability testing was subsequently performed using cycloheximide to inhibit protein translation. The protein stability of β-catenin in the shKIF18B group was significantly lower than that in the shCtrl group (**Figure 6B and 6C**). After cycloheximide treatment for 8 h, the β-catenin content of the shKIF18B group was approximately half that of the shCtrl group. We detected interactions between KIF18B and APC (**Figure 6D**). APC binds β-catenin and promotes its degradation. Notably, overexpression of KIF18B significantly blocked interactions between APC and β-catenin (**Figure 6D**). The protein stability of β-catenin in the shKIF18B-siAPC group (with knockdown of APC in the shKIF18B group) was recovered to a similar level as that in the shCtrl group (**Figure 6B and 6C**). Our collective results suggest that KIF18B competitively binds APC and indirectly regulates β-catenin protein levels.

### Knockdown of KIF18B suppresses tumor growth *in vivo*

**Figure 7:**
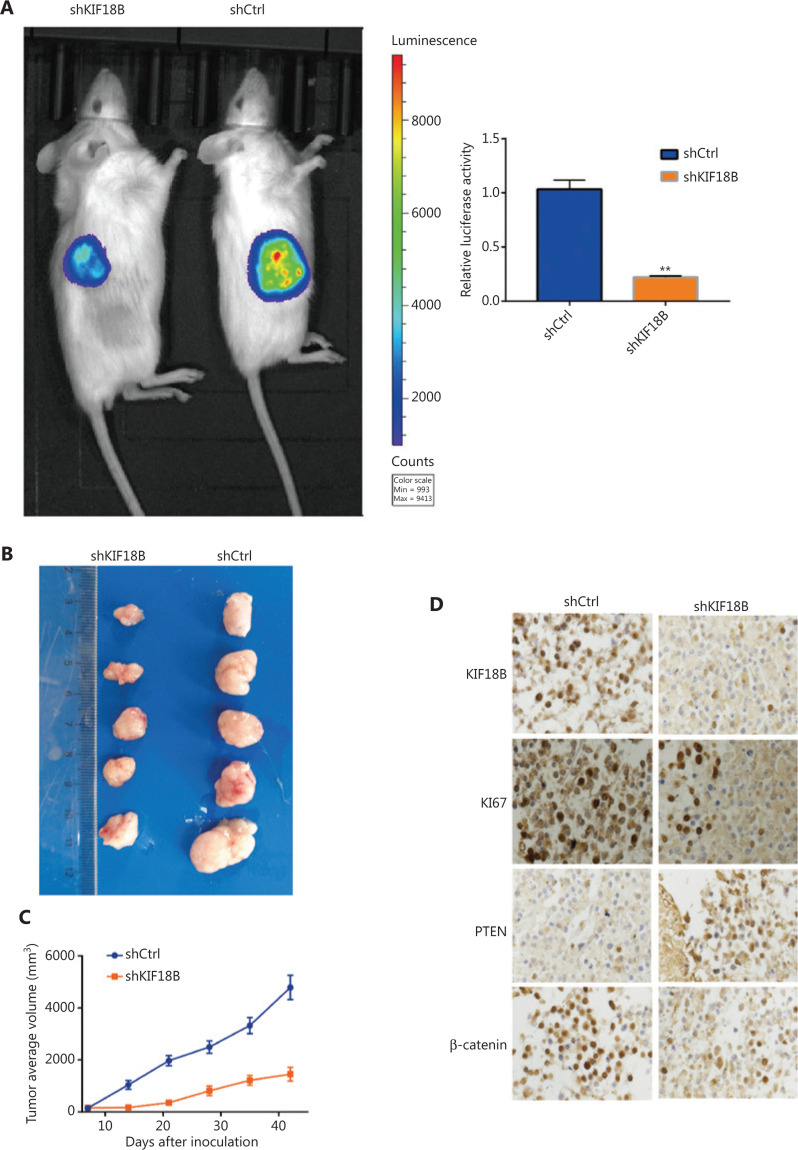
Knockdown of *KIF18B* suppresses tumor growth *in vivo*. (A) Luciferase activities of shKIF18B and shCtrl cells in mice intraperitoneally injected with luciferin. The luciferase activity of shKIF18B was 22.3% that of shCtrl (*n* = 3). Mann-Whitney U test was used for statistical analysis. (B) Tumor nodules from mice injected with shKIF18B and shCtrl cells. (C) Mean tumor volumes in mice treated with shKIF18B cells, compared with the control group (*n* = 8). Mice were implanted subcutaneously with 2 × 10^6^ shCtrl or shKIF18B cells and tumor volumes measured every 7 days. (D) Immunohistochemical staining for KIF18B, Ki67, PTEN, and β-catenin in tumor nodules of mice injected with shKIF18B and shCtrl cells (20 ×). Attenuated expression of Ki67, PTEN, KIF18B, and β-catenin was observed in the shKIF18B group.

A nude mouse xenograft model using U2OS cells was further established. The luciferase activity detected in mice injected with shKIF18B was significantly lower than that in mice injected with shCtrl cells (**Figure 7A**). Tumor volume and weight were lower in the shKIF18B than control group (**Figure 7B and 7C**). IHC staining revealed attenuated expression of Ki67, PTEN, KIF18B, and β-catenin in the sh-KIF18B group (**Figure 7D**), indicating that silencing of *KIF18B* inhibits tumor growth *in vivo*.

**Figure 8:**
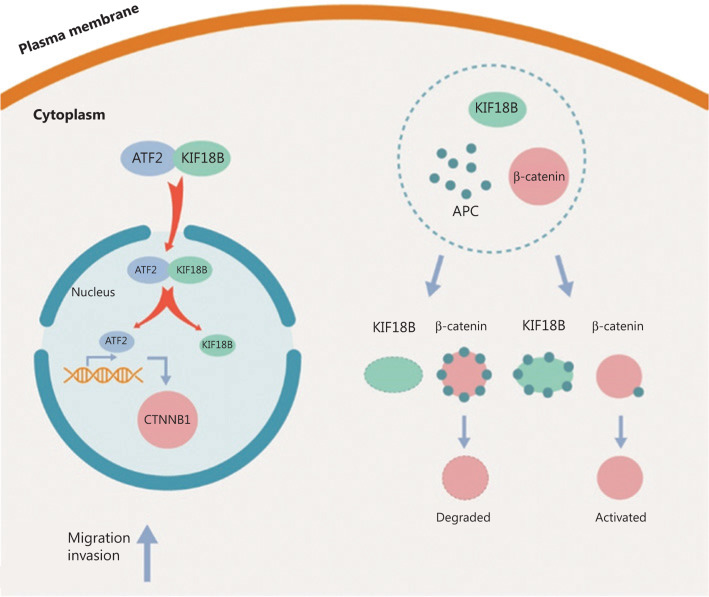
Schematic diagram of KIF18B-APC-β-catenin and KIF18B-ATF2-β-catenin pathways. ATF2 is transported into the nucleus and β-catenin mRNA expression is regulated by KIF18B. Simultaneously, β-catenin is protected from degradation through APC binding of KIF18B.

As shown in **Figure 8**, ATF2, the transcription factor, is transported into the nucleus by KIF18B, in turn, regulating β-catenin transcription. Simultaneously, β-catenin is protected from degradation *via* APC binding to KIF18B, which affects the β-catenin protein level.

## Discussion

KIF18B, a member of the kinesin family, plays an important role in cell division in a cell cycle-dependent manner^10^. Kinesin is a tetramer composed of two light chains and two heavy chains. Conformational changes occur in the neck region through binding and hydrolysis, and the two heads alternately bind and “walk” along microtubules. Transport bubbles or organelles comprising “cargo” bind kinesin tails and are transported to other locations^11^. Kinesin has been characterized as a microtubule-based molecular motor protein that participates in transport of vesicles, organelles, chromosomes, protein complexes, and binding proteins and is critical in intracellular material transport and cell division^15^. Decrease in KIF18A expression leads to G2 phase arrest, supporting a critical role in cell cycle regulation^28^. Several studies have shown that KIF18A is closely associated with breast, gastric, and kidney cancer^29–31^.

During mitosis, KIF18B interacts with EB1 to control the length of stellate microtubules^32^. Expression of KIF18B is upregulated in hepatocellular carcinoma^25^ and cervical cancer^26^. KIF18B has been shown to activate the Wnt/β-catenin signaling pathway in cervical cancer cells. *KIF18B* gene knockout induces cell cycle arrest and inhibits proliferation, migration, and invasion of cervical cancer cells. Conversely, overexpression of *KIF18B* promotes proliferation, migration, and invasion of cervical cancer cells^26^. In our study, the relative mRNA content in osteosarcoma clinical specimens was significantly higher than that in paracancerous tissues and the immortalized human osteoblast cell line, hFOB1.19. Protein and mRNA levels of *KIF18B* were additionally upregulated in the osteosarcoma cell lines HOS, U2OS, and Saos-2. Lentivirus-packaged shKIF18B was used to infect U2OS and HOS for construction of a shKIF18B cell line. Our experiments showed that proliferation of U2OS and HOS cells was significantly decreased with downregulation of KIF18B. In U2OS cells, KIF18B suppression induced G1 phase arrest, promoted apoptosis, and suppressed migration and invasion. Nude mice were inoculated with tumor cells transfected with shKIF18B and the effects of shKIF18B on tumorigenic ability observed. Tumor sizes in the shKIF18B group were significantly lower relative to that in the control group, suggesting that expression of KIF18B may be positively correlated with degree of osteosarcoma malignancy.

To clarify the specific mechanisms underlying the tumor-promoting effect of KIF18B in U2OS cells, microarray analysis was applied to detect the differences in transcription before and after *KIF18B* knockdown in U2OS cells. *CTNNB1*, a key gene involved in the classical Wnt pathway, was significantly downregulated with KIF18B suppression, as confirmed *via* qRT-PCR and Western blot. Additionally, c-MYC and cyclin D1, downstream regulators of CTNNB1 (β-catenin), were significantly downregulated. CTNNB1 overexpression led to recovery of the phenotype of shKIF18B cells, such as inhibition of proliferation, migration, and invasion. These results suggest that the function of KIF18B in osteosarcoma cells is closely associated with activation of the Wnt/β-catenin pathway. Several diseases, including malignant tumors, are associated with imbalances in the intracellular Wnt/β-catenin signaling pathway^33,34^. A host of important transcription and signal transduction factors are involved in regulation of the Wnt/β-catenin signaling pathway. β-Catenin, a marker of Wnt activity and a core factor transmitting Wnt signals, is highly expressed in almost all types of tumors. Consequently, the Wnt signaling pathway is abnormally activated, thus promoting cell colonization and migration, affecting the normal cell cycle and inducing tumor deterioration ^6,7,35^.

Knockout of *KIF18B* induced a decrease in β-catenin mRNA expression. Generally, β-catenin is thought to be regulated at the protein level, with limited reports of transcriptional regulation. To ascertain whether KIF18B regulates β-catenin transcription, we incorporated the promoter region of β-catenin into a luciferase reporter vector. Notably, luciferase activity was significantly decreased with knockdown of *KIF18B*. Data from ChIP assays additionally supported KIF18B activity on the promoter region of β-catenin. However, no studies to date have identified a DNA binding domain in KIF18B, indicating that interactions between KIF18B and the β-catenin promoter may be indirect. Further analyses revealed interactions between KIF18B and ATF2. With decreased KIF18B expression, distribution in the cytoplasm and nucleus was altered and the degree of nuclear aggregation of ATF2 was significantly decreased. Accordingly, we propose that KIF18B, a member of the driver protein family, regulates β-catenin at the transcriptional level by transporting the transcription factor, ATF2, into the nucleus. We additionally observed significantly lower β-catenin protein stability in the KIF18B knockout relative to the control group. The mechanism by which KIF18B affects the stability of β-catenin protein is yet to be established. Interestingly, we observed interactions between KIF18B and APC. In mammals, APC is expressed in most fetal tissues and adult epithelial cells. The *APC* gene encodes a multi-domain protein of 2843 amino acids. Mammalian APC contains an oligomerization domain, armadillo repeat domain, and 15 or 20 residue repeat domain, which is critical for binding to β-catenin^36,37^. Interestingly, an earlier report by Behrens et al.^38^ showed that β-catenin is downregulated *via* interactions with APC. Consequently, activation of the typical Wnt pathway triggered by APC inactivation could result in intestinal tumor occurrence. The protein stability of β-catenin in the shKIF18B-siAPC group was restored to that of the control group, compared with that in the shKIF18B group. APC inactivation is proposed to promote tumorigenesis through loss of cell adhesion^39,40^. Overexpression of KIF18B significantly hindered the interactions between APC and β-catenin, indicating that KIF18B competitively binds APC and protects β-catenin stability. The data support an indirect regulatory effect of KIF18B on β-catenin at the translational level.

## Conclusions

KIF18B is overexpressed in osteosarcoma cells and tissues and its expression is positively correlated with the degree of malignancy of U2OS cells. KIF18B promotes osteosarcoma cell proliferation *in vitro*. Downregulation of *KIF18B* leads to arrest of U2OS osteosarcoma cells in the G1/S phase, resulting in decreased proliferation, migration, and invasion. In addition, *KIF18B* knockdown suppresses expression of β-catenin, a key protein in the Wnt/β-catenin pathway, at the transcriptional level by decreasing the degree of nuclear aggregation. Simultaneously, KIF18B regulates β-catenin at the post-transcriptional level through interactions with the tumor suppressor gene *APC*. Based on the collective findings, we propose that KIF18B plays a carcinogenic role and may effectively serve as a novel prognostic biomarker of osteosarcoma.
