# Supplementary material for: KIF18B promotes tumor progression in osteosarcoma by activating β-catenin
Source: Cancer Biol Med. 2020 May 15;17(2):371–86. doi: 10.20892/j.issn.2095-3941.2019.0452 (PMC7309474; doi:10.20892/j.issn.2095-3941.2019.0452)
Supplement: Supplementary file 1 [file cbm-17-371-s001.pdf]

Supplementary materials

Table S1 Primers of genes for qRT-PCR

Gene	Left primer	Right primer
KIF18B	TTCACACCATTCTCCTGCCT	ATAAATGAGAGGGGAGGCCG
CTNNB	CTTACACCCACCATCCCACT	CCTCCACAAATTGCTGCTGT
WNT5A	CGTTAGCAGCATCAGTCCAC	ACGGCATCTCTCTTTCACCA
MYC	CTTTCCTCCACTCTCCCTGG	AACCCTCTCCCTTTCTCTGC
CCND1	TTTGTTGTGTGTGCAGGGAG	TTTCTTCTTGACTGGCACGC
ATF2	AGTACAAGTGGTCGTCGGAG	TGACTTCACTCTGCAGCTGA
CUX1	CTGACCACCTGCACAAGTTC	CAGCGGATAAGGGCAGTTTC
TCF3	GATGCTGCCTTTGGTCTCTG	CTCAGGTTTACACGGGGTCT
RUNX1	CCAGCAGAGGGGAGAAGAAA	ATGAAGCACTGTGGGTACGA
GATA1	TCTACCCTGCCTCAACTGTG	GAGTGATGAAGGCAGTGCAG
ACTB	CCCTGGAGAAGAGCTACGAG	CGTACAGGTCTTTGCGGATG

Table S2 Primers of CTNNB1 for the ChIP experiment (Figure 5B)

Primer	Left primer	Right primer
Primer-1	GGAATCAGTACCTGAAAACGCC	CCGTCTGTCACCATAGCACA
Primer-2	TCCGCCTGCATCTATGTGTT	GCAGCAAAATTGACGTTTGTCG
Primer-3	ACAGCGCTCTGGAGCTAATC	ATGGTGGATCCCACCTCTCA
Primer-4	AGGATACCAGGGCCACCTT	TATAAGTCGCGCAGAAGCCG

Table S3 Top 15 genes downregulated in the shKIF18B group

Entrez	Gene symbol	log FC	*P*
9315	NREP	−2.896	3.08652E-20
23234	DNAJC9	−2.683	2.99457E-20
10592	SMC2	−2.355	5.61178E-18
55026	TMEM255A	−2.245	6.16853E-13
57007	ACKR3	−2.181	7.06502E-15
692149	SCARNA14	−2.141	1.88317E-14
1499	CTNNB1	−2.080	1.18089E-15
26047	CNTNAP2	−2.008	6.80892E-16
55214	P3H2	−1.983	4.194E-13
25928	SOSTDC1	−1.956	1.36719E-15
4023	LPL	−1.950	2.88914E-15
81611	ANP32E	−1.946	9.50442E-22
54510	PCDH18	−1.925	1.21919E-14
23767	FLRT3	−1.884	1.26295E-12
7474	WNT5A	−1.862	1.12552E-21

CTNNB1 promoter

>FP005181 CTNNB1_1 :+U EU:NC; range -2000 to 100.

......ACTTATATAGTAAAATATCTTTAAGACAAAAATAAAAGTTATTTAAAGAATA
CGCTGGCCCTGAAACATGAGCTGTGCCTTAAACTACATATCCTACCTCCATGAG
TGAATGAATAAGTGGGTTTTCCGCCTGCATCTATGTGTTAAGACCTATTGTTTGC
TTGTATTAATTGTAGTCCTGGGATAAAAGCACAGAGGTAACTTTCACTGCTGCT
TTTTGTACTCTCTCCAATGTTTTGGAGGAAAAATAAGCACAAACAATAGCCTAG
ATF2 binding site → MUT GTTTGCAGTT
AGAAACTGAATCGATCATACTTGTTGCAGCTTCGACAAACGTCAATTTTGCTGC
ATTAGAATGGGAAACATTTTTCAGTCTATTGAAATGAATTACAAACGTTTTTAA
ATAGAAAATTAGTTAAAAAAATTGGAGGCTGCTTAATCGATAGCTTTCTCTATAA
ACATACTTGGATTTCACAAATAAGTAATACCGTAAAAATCTTCTTCTCCAAAGA
AAAATCCCCACAAATAAATCTATTGATACCTAGTGACAAG......

Figure S1 Substitution of the potential binding site of ATF2 at the promoter region of β-catenin with a mutant fragment.

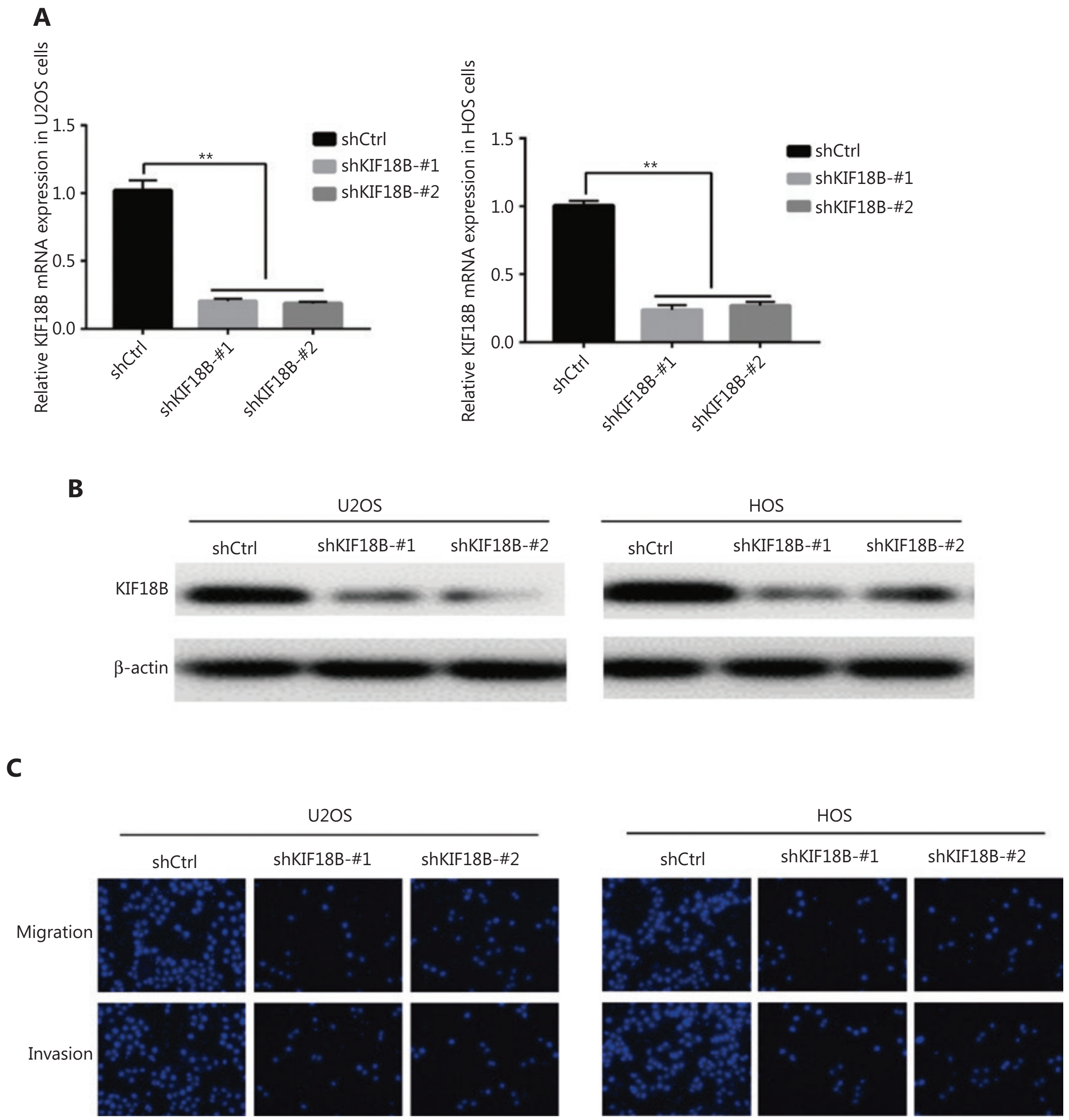

Figure S2 Knockdown of KIF18B affects migration and invasion of U2OS and HOS cell lines. mRNA (A) and protein (B) expression of KIF18B in U2OS and HOS cells. KIF18B mRNA and protein levels were significantly downregulated in all shKIF18B-transfected cell lines. (C) Transwell assays of shCtrl and shKIF18B cell lines. Knockdown of KIF18B impaired both cell migration and invasion abilities.
